# Inducible nitric oxide synthase regulates macrophage polarization via the MAPK signals in concanavalin A‐induced hepatitis

**DOI:** 10.1002/iid3.643

**Published:** 2022-06-06

**Authors:** Xiaoying Yao, Guiyuan Jin, Dong Liu, Xiaobei Zhang, Yonghong Yang, Yu chen, Zhongping Duan, Yanzhen Bi, Fenglian Yan, Yanli Yang, Hui Zhang, Guanjun Dong, Shanshan Li, Shumin Cheng, Huixin Tang, Feng Hong, Chuanping Si

**Affiliations:** ^1^ Medical Research Center, Affiliated Hospital of Jining Medical University Jining Shandong China; ^2^ Institute of Immune Precision Diagnosis and Therapy & Translational Medicine Affiliated Hospital of Jining Medical University Jining Shandong China; ^3^ Department of Clinical Laboratory Affiliated Hospital of Jining Medical University Jining Shandong China; ^4^ Fourth Liver Disease Center, Beijing YouAn Hospital Capital Medical University Beijing China; ^5^ Department of Infectious Disease Qingdao Municipal Hospital Qingdao Shandong China; ^6^ Institute of Immunology and Molecular Medicine Jining Medical University Jining Shandong China; ^7^ Department of Gastroenterology People's Hospital of Jia Xiang Jining Shandong China

**Keywords:** hepatitis, iNOS, macrophage, mitogen‐activated protein kinase

## Abstract

**Introduction:**

Acute liver inflammatory reactions contribute to many health problems; thus, it is critical to understand the underlying pathogenic mechanisms of acute hepatitis. In this study, an experimental in vivo model of concanavalin A (ConA)‐induced hepatitis was used.

**Materials and Methods:**

C57BL/6 (wild‐type, WT) or inducible nitric oxide synthase‐deficient (iNOS^−^^/−^) mice were injected with PBS or 15 mg/kg ConA via tail vein. Detection of liver injury by histological examination and apoptosis, and flow cytometry to detect the effect of immune cells on liver injury.

**Results:**

iNOS^−^^/−^ mice had lower levels of the liver enzymes aspartate aminotransferase and alanine aminotransferase, suggesting that they were protected against ConA‐induced pathological liver injury and that iNOS participated in the regulation of hepatitis. Furthermore, iNOS deficiency was found to lower CD86 expression and suppressed the messenger RNA levels of inflammatory factors in the liver. In vitro experiments also demonstrated that iNOS deficiency suppressed the sequential phosphorylation of the mitogen‐activated protein kinase pathway cascade, thereby inhibiting the M1 polarization of macrophages and consequently suppressing the transcription of inflammation factors.

**Conclusion:**

iNOS may contribute to ConA‐induced inflammation by promoting the activation of proinflammatory macrophages.

## Graphical abstract

We believe that our study makes a significant contribution to the literature because we identify that inducible nitric oxide synthase‐deficient may contribute to concanavalin A‐induced inflammation by promoting the activation of proinflammatory macrophages.

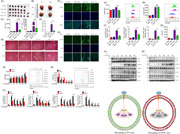

## 1 INTRODUCTION

Acute liver injury is a critical medical problem worldwide, characterized by a prompt defuctionalization of hepatocytes, which is a prerequisite for acute liver failure (ALF).^1^, ^2^ In 1992, it was shown that intravenous concanavalin A (ConA) administration could activate CD4^+^ T‐cells and macrophages in the liver of mice, resulting in the release of a large amount of inflammatory mediators and consequent cytokine‐dependent immune acute liver injury.^3^, ^4^, ^5^ In addition, activated T‐lymphocytes in the spleen release a large number of cytokines into circulation, which activated hepatic macrophages, further aggravating the liver injury. ConA‐induced hepatitis in mice mainly manifests as an elevation of transaminases along with severe liver damage, mimicking the features of viral hepatitis, autoimmune hepatitis, and other liver diseases mainly mediated by immune factors; thus representing a suitable experimental model for such human pathological conditions.

Specialized tissue‐resident macrophages can be classified as classical pathway activated or M1 macrophages and alternative pathway activated or M2 macrophages according to different cell surface specific phenotypes and functions. Normally, M1 macrophages have proinflammatory roles during liver injury, whereas M2 macrophages exert anti‐inflammatory effects during liver repair.^6^ In turn, macrophages colonizing different tissues can be divided into several subgroups based on their structural location and functional phenotype. Kupffer cells (KCs), a subtype of highly multifunctional macrophages, are present on the luminal side of the hepatic sinusoid, phagocytosing pathogens, and cellular debris.^7^, ^8^, ^9^ Previous studies show that M1‐polarized KCs secrete proinflammatory cytokines, such as tumor necrosis factor (TNF)‐α, interleukin (IL)‐12, reactive oxygen species, and inducible nitric oxide synthase, which can contribute to liver injury.^6^, ^10^ Injection of the ConA in mice rapidly induces intrahepatic interferon‐γ and TNF‐α, followed by tissue factor (TF), which aggravates fibrin deposition and liver necrosis. Studies suggest that these damages are obviously neutralized by pterostilbene (PTE) pretreatment. Upon further exploring the protective mechanism of PTE, researchers have found that PTE can reverse the ConA‐induced phosphorylation of signaling molecules, including the c‐Jun N‐terminal kinase (JNK), extracellular signal‐regulated kinase (ERK)1/2, p38, and p65.^11^

Nitric oxide (NO) is an important signaling molecule in immunological systems, involved in the regulation of diverse intra‐ and intercellular physiological and pathophysiological mechanisms.^12^, ^13^, ^14^, ^15^ Overproduction of NO is cytotoxic and has multiple roles in innate and adaptive immune responses. In mammals, NO production is catalyzed by a family of NO synthases, comprising three distinct isoforms: neuronal NOS (nNOS), inducible nitric oxide synthase‐deficient (iNOS), and endothelial NOS (eNOS). nNOS and eNOS are Ca^2+^‐dependent enzymes,^16^, ^17^ whereas iNOS is a Ca^2+^‐independent enzyme.^17^, ^18^ iNOS is mainly expressed in macrophages and neutrophils,^19^ and is highly upregulated in activated murine macrophages.^20^ Our present study, in vivo and vitro, demonstrated that iNOS deficiency prevents ConA‐induced liver injury by regulating the activation of macrophages and the phosphorylation levels of the mitogen‐activated protein kinase (MAPK) pathway.

## 2 MATERIALS AND METHODS

### 2.1 Mice

C57BL/6 mice were purchased from Peng Yue experimental animal breeding company. iNOS‐deficient (iNOS^−^^/−^) mice were kindly provided by professor Tang Hua from Shandong First Medical University Medical College. C57BL/6 and iNOS^−^^/−^ mice (male, 6−8 weeks) were bred in specific pathogen‐free conditions with free access to food and water. 

### 2.2 Murine model of ConA acute liver injury

C57BL/6 or iNOS^−^^/−^ mice were respectively injected with ConA (15 mg/kg) via tail vein to establish the mice model of acute liver injury. The mice injected with the same volume of phosphate buffered solution (PBS) were taken as the controls.

### 2.3 Preparation of peritoneal macrophages

One milliliter of 6% starch broth by intraperitoneal injection of C57BL/6 or iNOS^−^^/−^ mice. After 72 h, abdominal cavity cells were repeatedly washed with PBS and placed in a 15 ml centrifuge tube. These cells were planted on the Dulbecco's modified eagle medium subsequently. Adherent cells were used in subsequent experiments after 6 h. The adherent macrophages were treated with ConA (0, 25, 50, 100, 200, and 400 µg/ml) for 30 min, 1, 6, or 24 h.

### 2.4 Flow cytometry

Cells (1 × 10^6^) were stained with fluorochrome‐conjugated mouse antibodies in PBS for 30 min at 4°C according to the manufacturer's instructions. Antibodies were purchased from eBioscience. Cells were analyzed by Becton Dickinson Verse.

### 2.5 Quantitative real‐time PCR (RT‐PCR) (quantitative RT‐PCR [qRT‐PCR])

Complementary DNA was prepared using TRIzol and HiScript® III RT SuperMix for qPCR (+gDNA wiper) from Vazyme Biotech according to the manufacturer's instructions. qRT‐PCR was done using SYBR Green PCR Master Mix (Vazyme Biotech). The following sequence‐specific primers were synthesized. Sequences were shown in supplemental data (Supporting Information: Table S1).

### 2.6 NO production

Diluted the standard with the solution used for the sample and added Griess Reagent I and Griess Reagent II (Beyotime Biotechnology) in sequence. The absorbance was measured at 540 nm using an enzyme‐labeled instrument (BioTek).

### 2.7 Western blot analysis

Total protein was prepared using radio immunoprecipitation assay buffer to analyze the phosphorylation level of signaling pathway proteins. The protein was resolved using 10% sodium dodecyl sulfate‐polyacrylamide gel electrophoresis followed by transfer onto polyvinylidene fluoride membranes (Millipore) for 2 h with a standard transfer solution. Membranes were incubated with primary antibodies for p38, p‐p38, JNK, p‐JNK, ERK1/2, and p‐ERK1/2 antibodies at 4°C overnight after being blocked with 3% BSA. Horseradish peroxidase‐labeled goat anti‐rabbit (Abclonal) was used as the secondary antibody. β‐Actin was chosen as the internal control.

### 2.8 Histopathology

Sections were cut from paraffin‐embedded liver tissues and stained with hematoxylin and eosin. Terminal deoxynucleotidyl transferase‐mediated uridine triphosphate nick‐end labeling assay was strictly executed according to the manufacturer's protocol.

### 2.9 Statistical analysis

All experimental data were obtained from at least three independent experiments. All data are normally distributed continuously and expressed as the mean ± standard error of the mean. Statistical analysis was performed using analysis of variance followed by the Bonferroni test for individual comparisons between group means. A two‐sided *p *< .05 was considered to be significant.

## 3 RESULTS

### 3.1 iNOS deficiency can mitigate ConA‐induced ALF

**Figure 1:**
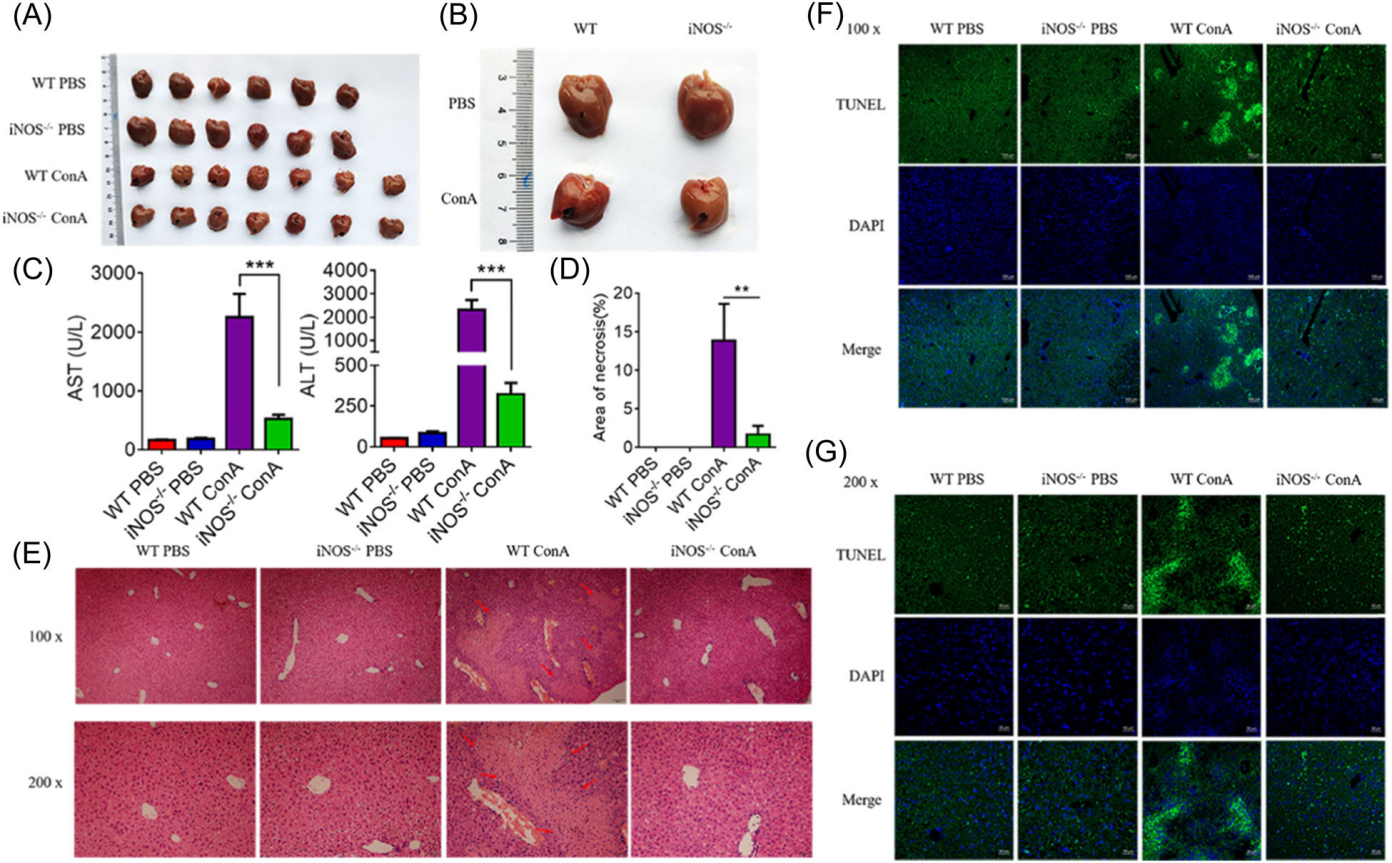
iNOS deficiency can mitigate ConA‐induced ALF. Six to 8‐week‐old C57BL/6 or iNOS^−^^/−^ mice were intravenously injected with PBS or ConA (15 mg/kg of body weight) for 12 h to establish the liver injury model. (A and B) The images of the liver showed that the sizes were smaller and the textures were rougher in the liver of WT mice. (C) The biochemical evaluations of ALT and AST in the serum of mice were detected. (D) The necrotic area stained with hematoxylin and eosin and used for the liver sections was analyzed with Image‐Pro Plus (magnification, ×100). (E) Tissues of the liver were fixed with 4% paraformaldehyde and paraffin‐embedded liver sections were stained with hematoxylin and eosin (flaky necrosis indicated by the red‐pointed tip). (F and G) Apoptosis in the cells of the liver was analyzed by terminal deoxynucleotidyl transferase‐mediated uridine triphosphate nick‐end labeling. Data shown are representative of three independent experiments. Error bars represent the standard error of the mean (SEM). **p < .01, ***p < .001. ALF, acute liver failure; ALT, alanine aminotransferase; AST, aspartate aminotransferase; iNOS, inducible nitric oxide synthase‐deficient; SEM, standard error of the mean; WT, wild‐type.

To elucidate the effect of iNOS deficiency on ALF, a mouse model of ConA‐induced ALF was used. The sizes and the textures of the liver were more anomalous in wild‐type (WT) mice compared to iNOS^−^^/−^ mice after administration of ConA (Figure 1A,B). The levels of aspartate aminotransferase (AST) and alanine aminotransferase (ALT) were determined, revealing that were markedly reduced in iNOS^−^^/−^ mice compared with WT mice, which suggests that iNOS^−^^/−^ mice were resistant to ConA‐induced liver injury (Figure 1C). Pathological tissue sections also showed that liver injury in WT mice was much more severer (Figure 1D,E), with flaky necrosis (indicated by the red‐pointed tip, Figure 1E) compared to slight punctate hepatocytes necrosis observed in iNOS^−^^/−^ mice. Consistent with the histopathological results, terminal deoxynucleotidyl transferase‐mediated uridine triphosphate nick‐end labeling staining (Figure 1F,G) showed greater apoptotic area and severer injury in the liver of WT mice. Altogether, these findings suggest that iNOS mediates the occurrence and development of ALF.

### 3.2 iNOS deficiency inhibits ConA‐induced proinflammatory macrophage activity in vivo

**Figure 2:**
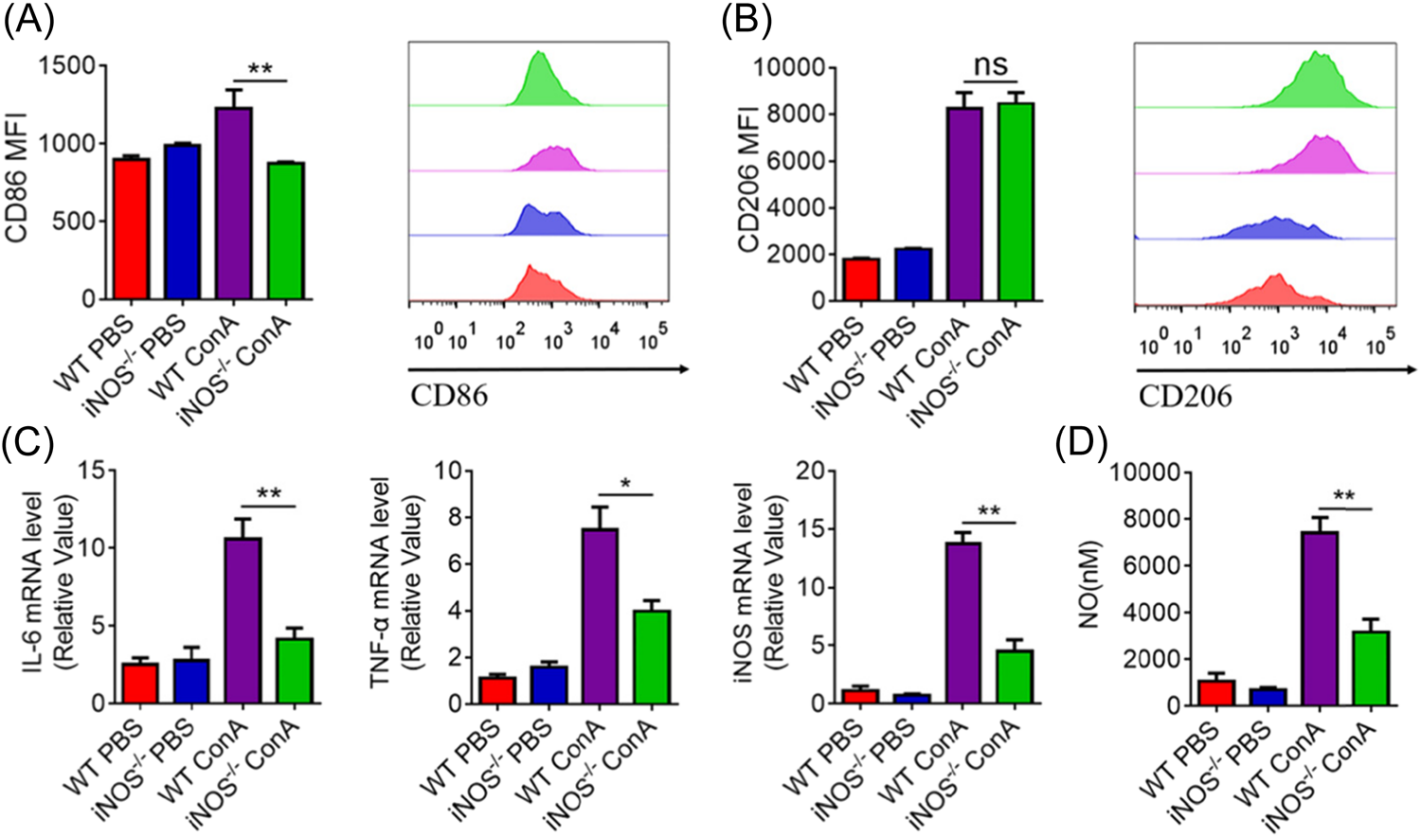
iNOS deficiency inhibits ConA‐induced proinflammatory macrophage activity in vivo C57BL/6 mice or iNOS^−^^/−^ mice were treated with PBS or ConA (15 mg/kg of body weight). FACS analysis was performed to assess the expressions of CD86 (A) and CD206 (B) in the liver after 12 h. (C)The expressions of IL‐6, TNF‐α, and iNOS in the liver were examined by qRT‐PCR. Data shown are representative of three independent experiments. Error bars represent the standard error of the mean (SEM). (D) Nitric oxide production was determined by the Griess reagent method in the liver after 12 h. *p < .05, **p < .01; ns denotes *p* > .05. IL,interleukin; iNOS, inducible nitric oxide synthase‐deficient; qRT‐PCR, qualitative real‐time PCR; SEM, standard error of the mean.

High levels of iNOS expression contribute to, the secretion of large amounts of NO by macrophages to cause ConA‐related inflammation.^21^ To explore the reduced liver injury in iNOS^−^^/−^ mice is dependent on macrophages, distributions of M1 and M2 macrophages in the liver, indicated by the surface markers of CD86 and CD206, respectively, were evaluated by flow cytometry. The mean fluorescence intensity of CD86 in the liver of WT mice was higher than that in iNOS^−^^/−^ mice, while the expression of CD206 was not significantly different between the two groups (Figure 2A, B). However, the expression of IL‐6, TNF‐α, and iNOS messenger RNA (mRNA) were remarkably enhanced in the liver of WT mice (Figure 2C). Furthermore, the production of NO in the liver was obviously elevated in WT mice than in iNOS^−^^/−^ mice (Figure 2D). Thus, the reduction of liver damage in iNOS^−^^/−^ mice may be due to decreased M1 macrophage polarization and the production of NO.

### 3.3 iNOS deficiency inhibits ConA‐induced proinflammatory macrophage activity in vitro

**Figure 3:**
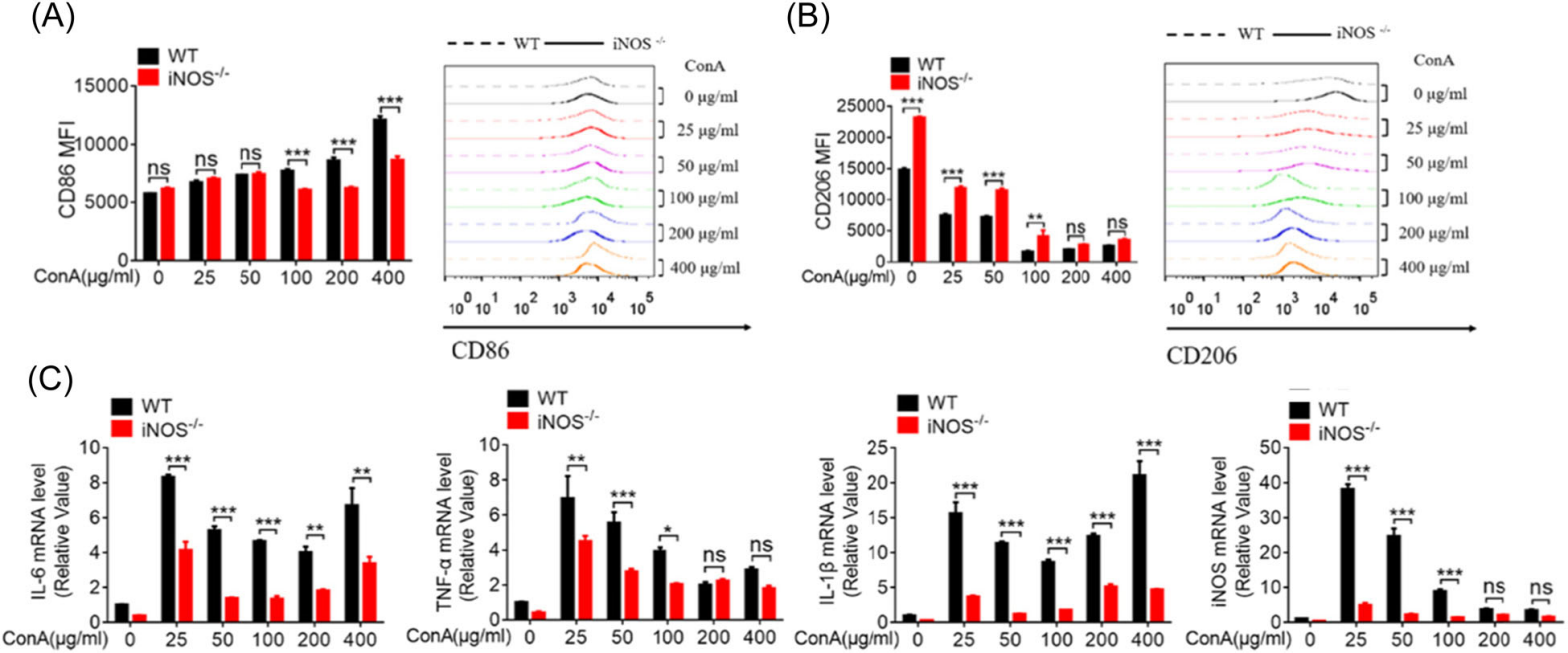
iNOS deficiency inhibits ConA‐induced proinflammatory macrophage activity in vitro peritoneal macrophages were treated with ConA (0, 25, 50, 100, 200, and 400 μg/ml) for 24 h. FACS analysis was performed to assess the expressions of CD86 (A) and CD206 (B) after 24 h. (C) Expressions of IL‐6, TNF‐α, IL‐1β, and iNOS were measured using qRT‐PCR after 6 h. Data shown are representative of three independent experiments. Error bars represent the standard error of the mean (SEM). *p < .05, **p < .05, ***p < .01; ns denotes *p* > .05. IL, interleukin; iNOS, inducible nitric oxide synthase‐deficient; qRT‐PCR, qualitative real‐time PCR; SEM, standard error of the mean.

The knockout of iNOS in the iNOS^−^^/−^ mice was verified using western blot analysis (Supporting Information: Figure S1). In subsequent cellular experiments, we investigated the effect of ConA on its activation in primary peritoneal macrophages of mice. Next, primary peritoneal macrophages were treated with increasing concentrations of ConA in vitro to explore its effect on macrophage activation. Flow cytometry analysis showed that ConA could promote the upregulation of CD86 in peritoneal macrophages to a greater extent in WT than in iNOS^−^^/−^ cells at 100, 200, and 400 μM (Figure 3A). Moreover, the total amount of CD206 cells was drastically reduced and the expression of CD206 was no different at 100, 200, and 400 μM (Figure 3B). Similarly, the relative expression of several inflammatory factors, such as IL‐6, TNF‐α, IL‐1β, and iNOS, was significantly higher in WT than in iNOS^−^^/−^ macrophages (Figure 3C). These results seem to suggest that the difference in the inflammatory reaction of M1 macrophages with or without iNOS deletion plays an important regulatory role in the inflammatory response elicited by ConA.

### 3.4 iNOS deficiency inhibits the MAPK signaling pathway

**Figure 4:**
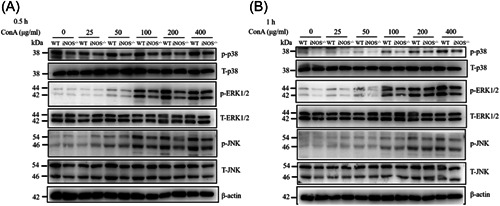
iNOS deficiency inhibits the MAPK signaling pathway peritoneal macrophages were treated with ConA (0, 25, 50, 100, 200, and 400 μg/ml) for 0.5 h (A) and 1 h (B). Cell lysates were prepared and subjected to immunoblotting with the indicated antibodies. β‐Actin was chosen as a loading control. iNOS, inducible nitric oxide synthase‐deficient; MAPK, mitogen‐activated protein kinase; SEM, standard error of the mean.

**Figure 5:**
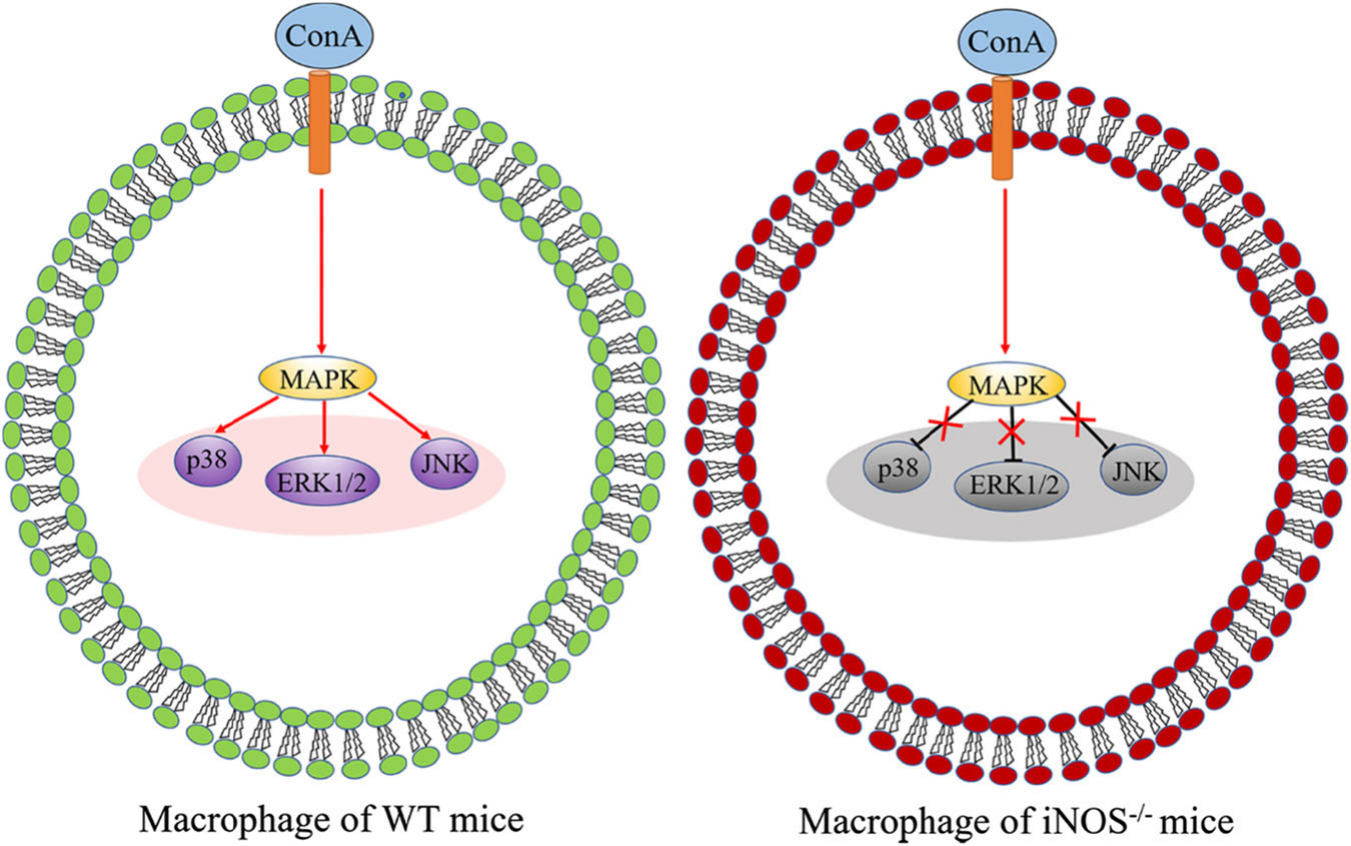
The regulatory mechanism of iNOS on the activation of MAPK pathway. This schematic shows that the deficiency of iNOS decreased the phosphorylation of p38, ERK1/2, and JNK, and further inhibited the activation of the MAPK signaling pathway. iNOS, inducible nitric oxide synthase‐deficient; JNK, c‐Jun N‐terminal kinase; MAPK, mitogen‐activated protein kinase; SEM, standard error of the mean; WT, wild‐type.

The MAPK signaling pathways play important roles in regulating hepatic inflammatory responses.^22^, ^23^ Thus, ConA effects on the activation of the MAPK pathways and its downstream targets in the presence or absence of iNOS were evaluated. To test this, peritoneal macrophages were treated with different concentrations of ConA or vehicle control for 0.5 or 1 h, and then collected for western blot analysis assessment. As shown in Figures 4A,B, ConA induced phosphorylation of p38, ERK1/2, and JNK at both time points, an effect that was suppressed in the absence of iNOS, indicating that iNOS positively regulates ConA‐induced activation of the MAPK pathway. Taken together, the deficiency of iNOS inhibits the activation of MAPK pathways, thereby protecting against ConA‐induced inflammation (Figure 5).

## 4 DISCUSSION

This study aimed to investigate whether iNOS deficiency could affect the occurrence and development of ConA‐induced acute liver injury by regulating macrophage polarization. The results of the study revealed that iNOS^−^^/−^ mice had significantly reduced serum levels of AST and ALT and that the area of liver necrosis and the degree of apoptosis were reduced compared to WT mice. Moreover, in vitro experiments revealed that iNOS deficiency suppresses the phosphorylation of MAPK pathway proteins. Collectively, this study demonstrates that iNOS deficiency can inhibit ConA‐induced polarization of macrophages and the production of inflammatory factors.

ConA is, a lectin also known as a phytohemagglutinin, that acts as an activator of the immune system.^1^, ^24^ The ConA‐derived mouse model of liver injury has important cellular and molecular similarities with several human immune‐mediated disorders, such as acute viral hepatitis, autoimmune hepatitis, or liver damage caused by drug toxicity. Therefore, it has become a classic research model for researchers to study immune‐mediated liver damage mechanisms. Upon administration, ConA first binds to the mannose on the surface of the sinusoidal endothelial cells (SECs), which leads to the rupture of the SEC membrane, formation of follicles, and disruption of the cytoplasm. Subsequently, ConA separates from the SECs and then binds to KCs. CD4^+^ T‐cells then recognize the T‐cell receptor and major histocompatibility complex class II molecules on KCs modified by ConA and become activated.^25^

ConA‐induced hepatitis is mediated by T cell‐derived interferon‐γ and KC‐deriver TNF‐α, causing massive liver necrosis with dense infiltration of leukocytes and release of the ALT and AST from the cytoplasm of hepatocytes into the blood.^26^, ^27^, ^28^ Macrophages play a bridging role in ConA‐mediated immune liver injury; therefore, this study was focused on exploring the role of macrophages. Tsutsui et al.^25^ demonstrated that KCs play a pivotal function in *Propionibacterium acnes*‐induced lipopolysaccharides sensitization and ConA‐induced severe hepatitis by activating hepatic granuloma formation and TF expression, thereby resulting in fibrin formation and platelet activation. In contrast, others reported that KCs negatively regulate acetaminophen‐induced acute liver injury.^29^, ^30^, ^31^ The present study, revealed that macrophages are preferentially polarized toward an M1 phenotype in WT mice compared to iNOS^−^^/−^ mice. However, this phenomenon is not observed in PBMCs and splenocytes. In addition, proinflammatory factors such as IL‐6, TNF‐α, and iNOS are notably higher in the livers of WT than in iNOS^−^^/−^ mice.

In vitro experiments with mouse peritoneal macrophages demonstrated that the expression of CD86, an M1 macrophage marker, was not significantly different between WT and iNOS^−^^/−^ cells exposed to 25 and 50 µM ConA, but was significantly reduced in the iNOS‐deficient macrophages compared to the WT macrophages at 100, 200, and 400 μM. Additionally, the expression of CD206, which represents M2 macrophage polarization, was found to be higher in the iNOS^−^^/−^ macrophages at 0, 25, 50, and 100 μM ConA, but nonsignificant trend, compared to the WT macrophages at 200 and 400 μM. Overall, with or without iNOS deficiency, the activation of M2 macrophage generally decreased with increasing ConA concentration, while the activation of M1 macrophage dominated in the inflammatory response. Concurrently, the mRNA levels of IL‐6, TNF‐α, IL‐1β, and iNOS were significantly upregulated in WT macrophages, indicating that ConA induces M1 macrophage polarization in an iNOS‐dependent manner.

Numerous studies have shown that the MAPK signaling pathways are widely involved in the immune response of macrophages.^21^, ^32^, ^33^, ^34^ Herein it was found that iNOS deficiency can inhibit the ConA‐induced phosphorylation of p38, ERK1/2, and JNK, suggesting that iNOS deficiency may directly suppress the activation of ConA‐induced downstream signaling pathways in macrophages. The protective effects of iNOS deletion in the ConA‐induced hepatitis model may be therefore related to reductions in MAPK phosphorylation and consequent release of downstream inflammatory factors. However, our study has limitations to a certain extent, further studies are required to elucidate its specific mechanism. Taken together, iNOS regulates the stimulation of the immune cells during ALF, the definite mechanism of which needs more investigation. So, more in‐depth research is needed to explore its mechanism.

## AUTHOR CONTRIBUTIONS

This study was conceived by Chuanping Si and Feng Hong​​​​​. Xiaoying Yao, Guiyuan Jin, Dong Liu, Xiaobei Zhang, Yonghong Yang, Yanzhen Bi, Fenglian Yan, and Yanli Yang performed experiments in the current study. Hui Zhang, Guanjun Dong, Shanshan Li, and Shumin Cheng provided experimental assistance. Xiaoying Yao and Huixin Tang wrote the original draft. Yu chen, Zhongping Duan, Feng Hong, and Chuanping Si reviewed the manuscript.

## CONFLICT OF INTEREST

The authors declare no conflict of interest.

## ETHICS STATEMENT

This study was approved by the Institutional Animal Care and Use Committee of Jining Medical University Affiliated Hospital, China (Reference number 2021C063).

## Supporting information

Supporting information.
