# Supplementary material for: Inducible nitric oxide synthase regulates macrophage polarization via the MAPK signals in concanavalin A‐induced hepatitis
Source: Immun Inflamm Dis. 2022 Jun 6;10(7):e643. doi: 10.1002/iid3.643 (PMC9168548; doi:10.1002/iid3.643)
Supplement: Supplementary file 1 — Supporting information. [file IID3-10-e643-s001.docx]

**Supplemental Data**

**Table 1** Quantitative real-time RT–PCR primer sequences

| Gene | Forward Primer | Reverse Primer |
| --- | --- | --- |
| IL-β | 5′-GAAATGCCACCTTTTGACAGTG-3′ | 5′-TGGATGCTCTCATCAGGACAG-3′ |
| IL-6 | 5′-CCAGAAACCGCTATGAAGTTCCT-3′ TATGAAGTTCCT-3′ TATGAAGTTCCT-3′ TATGAAGTTCCT-3′ | 5′-CACCAGCATCAGTCCCAAGA-3′ |
| TNF-α | 5′-GCCACCACGCTCTTCTGTCT-3′ | 5′-GGTCTGGGCCATAGAACTGATG-3′ |
| iNOS | 5'-CTGCAGCACTTGGATCAGGAACCTG-3' | 5'-GGAGTAGCCTGTGTGCACCTGGAA-3' |
| β-actin | 5′-GTGACGTTGACATCCGTAAAGA -3′ | 5′-GCCGGACTCATCGTACTCC -3′ |

The 2^-ΔΔCt^ method was used for real-time quantitative PCR gene expression analysis. All quantification data were presented as a ratio to the β-actin level.


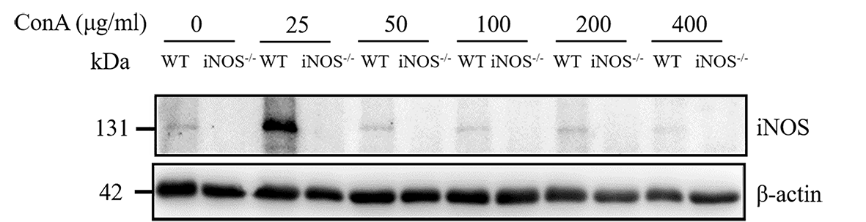


**FIGURE S1.** Peritoneal macrophages were treated with ConA (0, 25, 50, 100, 200, and 400 μg/ml) for 24 h. Cell lysates were prepared and subjected to immunoblotting with the indicated antibodies. β-actin was chosen as loading control.
